# Clinico-epidemiological profile of molar pregnancies in a tertiary care centre of Eastern Nepal: a retrospective review of medical records

**DOI:** 10.1186/s40661-015-0017-y

**Published:** 2015-10-31

**Authors:** Nimisha Agrawal, Reshu Agrawal Sagtani, Shyam Sundar Budhathoki, Hanoon P. Pokharel

**Affiliations:** Department of Obstetrics and Gynaecology, B.P. Koirala Institute of Health Sciences, Dharan, Nepal; School of Public Health and Community Medicine, B.P. Koirala Institute of Health Sciences, Dharan, Nepal

**Keywords:** *Molar pregnancy*, *Hydatidiform mole profile*, *Retrospective*, *Nepal*

## Abstract

**Background:**

The incidence of molar pregnancy has demonstrated marked geographic and ethnic differences. The reported data in Nepal is inconsistent with minimal published literature. Thus, we designed a study to determine prevalence of molar pregnancies and demonstrate clinical and epidemiological characteristics of the patients attending a tertiary care center in eastern Nepal.

**Methods:**

A retrospective review of medical records was conducted to determine the prevalence of molar pregnancies at the B.P. Koirala Institute of Health Sciences (BPKIHS) from the year 2008 to 2012. Secondary data from the medical records were analyzed. Annual and 5-year prevalence of molar pregnancy per 1000 live births was calculated. Demographic characteristics, clinical presentation, management methods and complications of molar pregnancy were studied.

**Results:**

The 5- year prevalence of molar pregnancy at BPKIHS is 4.17 per 1000 live births with annual prevalence ranging 3.8–4.5 per 1000 live births. More than one third of the patients were in the age group of 20–35 years and majority of them were of Hindu religion. For more than one third (41.7 %) of the patients, it was their first pregnancy while about 10 % gave a positive past history of molar pregnancy. Abnormal uterine bleeding (86.3 %) was the most frequent complaint, suction evacuation was the most common method of treatment and more than half of the patients required prolonged care after initial management.

**Conclusion:**

There is a need for studies at country level which will give us a national figure on molar pregnancies. Thus, a standardized clinic-epidemiological profile of molar pregnancy in Nepal can be created.

## Background

The management of gestational trophoblastic disease (GTD) depicts one of the success stories of modern medicine. As the majority, if not all, GTDs are potentially curable with the retention of reproductive function, once the correct diagnosis is made and treatment is commenced early enough [1–4] GTD constitutes a spectrum of tumors and tumor-like conditions characterized by abnormal proliferation of pregnancy associated trophoblastic tissues of varying propensities for invasion and spread [4–7].

They include complete and partial hydatidiform mole, invasive mole, placental-site trophoblastic tumor (PSTT), and choriocarcinoma. Hydatidiform mole is the most common GTD [5, 7].

The incidence of GTD varies greatly in different parts of the world, with 0.4 per 1000 birth in United States of America to 12.5 per 1000 births in Taiwan [8]. In Nepal, hospitals in Kathmandu valley have recorded its incidence as 5.1, 2.9, 2.8, and 4.1 per 1000 live births [9]. These 10–20‐fold variations in the incidence of molar pregnancy might be overestimated by reporting biases, such as population‐based and hospital‐based data [2]. Published literature on incidence of hydatidiform mole from this part of the world is rather minimal.

Maternal age is the most consistent risk factor for GTD in geographical regions and ethnic groups. Commonly affecting women in the reproductive age group, GTD has the propensity to become malignant but relatively easy to identify, diagnose and treat. Clinically hydatidiform mole presents with amenorrhoea, painless vaginal bleeding and spontaneous passage of grape-like vesicles, high serum and urinary β human chorionic gonadotrophin (βHCG) levels. There may also be hyperemesis gravidarum, doughy uterus, inappropriate uterine size, bilateral theca lutein cyst and rarely, features of thyrotoxicosis and pre-eclampsia in the first half of pregnancy [10–12]. Hydatidiform mole is a relatively common gynecological problem which could present like spontaneous abortion, one of the most common gynecological emergencies. Ultrasonography is a simple non-invasive examination which can correctly identify the placental molar transformations in-utero. Currently with widespread use of first trimester ultrasonography a significant proportion of patients with molar pregnancy are asymptomatic at the time of diagnosis. Careful and reliable human chorionic gonadotropin monitoring is essential for the early detection of post molar persistent gestational trophoblastic tumor.

Studies concerning the epidemiological characteristics, clinical presentation, management practices and outcome of molar pregnancy are rarely published from Nepal apart from a few case reports. Considering the relative lack of published epidemiology studies on hydatidiform mole from Nepal and considering the varied incidence rates reported from Asian countries, there is a need to determine the incidence rate in eastern Nepalese population in a well designed epidemiological study. With this background, we analyzed the past medical records of 5 years to determine the prevalence of molar pregnancy, evaluate the management practices and determine the outcome of hydatidiform mole at B P Koirala Institute of Health Sciences (BPKIHS), a tertiary care centre in eastern part of Nepal.

## Methods

A retrospective review of medical records was conducted at B. P. Koirala Institute of Health Sciences (BPKIHS) to develop a clinio—epidmiological profile of molar pregnancy. All women who are diagnosed with molar pregnancy/ hydatidiform mole (complete or partial) sonologically or histopathologically, and reporting to BPKIHS for treatment during the study period i.e. between 2008–2012 were included in the study. The data for total deliveries conducted in the hospital during the study period was also taken from the hospital registries. In-patient medical records from the medical records section of BPKIHS from the year 2008 to 2012 were reviewed and information of the current study was acquired.

The current study was approved by the Institutional Ethical Review Board (IERB), BPKIHS. Permission from the Hospital authority was taken to access the medical records was taken. All information collected from the hospital records were kept confidential. The details of maternal characteristics like maternal age, parity and period of gestation at the time of presentation, clinical presentation, diagnostic tools, management and complications was noted for each of cases. Details of past obstetric history such as previous history of molar pregnancy was noted as well as complications of molar pregnancy occurring during the hospital stay, need for blood transfusion or intensive care unit stay was noted. Anemia is classified according to the WHO criteria for anemia during pregnancy [13].

Descriptive statistics were used to summarize the continuous and categorical variables. Period prevalence was calculated by the number of cases of molar pregnancies reported to the institution for treatment during the study period for every 1000 live births delivered during the same period. All statistical analyses were performed using SPSS version 11.5.

## Results

A total of 48,805 live births took place in BPKIHS during from 2008 and 2012 with 204 molar pregnancies. The 5- year prevalence of molar pregnancy at BPKIHS is 4.17 per 1000 live births. The annual prevalence of molar pregnancies during the study period ranged from 3.8–4.5 per 1000 live births as shown in Table 1.

**Table 1:** Prevalence of molar pregnancy per 100 live births at BPKIHS, Nepal

Year	No. of molar pregnancies/year	Percentage (%)	Number of live births/year	Prevalence/1000
2008	38	18.6	8976	4.2/1000
2009	38	18.6	9866	3.8/1000
2010	42	20.6	10234	4.1/1000
2011	44	21.6	9651	4.6/1000
2012	42	20.6	10078	4.2/1000
Total	204	100.0	48805	4.2/1000

Table 2 shows the clinical profile of the patients reporting to BPKIHS for treatment of molar pregnancy. A total of 204 cases of molar pregnancy were included in this study. More than one third of the patients were in the age group of 20–35 years with a range of 16–51 and mean age 23.9 years. Majority of the patients belonged to Hindu religion. More than one third (41.7 %) of the patients were primigravida and about ten per cent gave a positive past history of molar pregnancy. The period of gestation ranged from 8 to 34 weeks with a major proportion (66.4 %) having period of gestation more or equal to 13 weeks with average uterine height of 17.8 weeks. About a quarter (24.5 %) of the patients gave a positive history of contraceptives with injectables (66 %) being the most frequently used followed by oral contraceptives (26 %). More than one third (38.7 %) of patients had pallor on examination. The Ultra-sonography (USG) findings suggest that more than one fifth (20.6 %) of pregnant women had theca lutein cysts. The blood grouping found A positive to be the most common blood group followed by O positive and B positive.

**Table 2:** Clinico-epidemiological profile of patients with molar pregnancy attending BPKIHS, Nepal

Variables	Categories	Number	Percentage
Age group in years	≤20	51	25.0 %
	20–35	143	70.1 %
	≥35	10	04.9 %
Mean age ± sd = 23.9 ± 6.6 Range = 16–51 years			
Religion	Hindu	174	85.8 %
	Kirat	24	11.8 %
	Buddhist	03	01.5 %
	Muslim	02	01.0 %
Parity	Primigravida	85	41.7 %
	Multigravida	119	58.3 %
Previous history of molar pregnancy	Yes	18	08.8 %
	No	186	91.2 %
Period of gestation	≥13 weeks	131	64.2 %
	<13 weeks	73	35.8 %
Period of gestation (in weeks) Mean age ± sd = 15.9 ± 5.8 weeks; Range = 6–34 weeks			
Previous history of contraceptive usage	Yes	50	24.5 %
	No	154	75.5 %
Type of contraceptive use	Oral contraceptives	13	26.0 %
	Injectable (Depo)	33	66.0 %
	IUCD (Copper-T)	03	06.0 %
	Intra dermal patch(Norplant)	01	02.0 %
Mean ± Sd (uterine size in weeks) 17.8 ± 6.5 weeks; Range: 06–36 weeks			
Pallor	Presence	79	38.7 %
	Absence	125	61.3 %
Theca lutein cyst on USG	Presence	42	20.6 %
	Absence	162	79.4 %
Blood group	A positive	80	39.2 %
	O positive	63	30.9 %
	B positive	52	25.5 %
	AB positive	07	03.4 %
	B negative	02	01.0 %

The hCG level of patients on an average was 18644.70 IU/ml while post management during the follow up after 1 week was 4156.84 IU/ml.

There were 28 (13.7 %) patients who had come to our hospital for regular ANC checkup, who were later diagnosed molar pregnancy. The rest 176 (86.3 %) cases presented with one or more complaints. Abnormal uterine bleeding (86.3 %) was the most frequent complaint of the patients coming to our center with molar pregnancy followed by, pain per abdomen (33.8 %), hyper emesis (26.5 %) and passage of grape like cysts (11.8 %). The various treatment modalities for molar pregnancy were used. Suction evacuation was the most common method of treatment followed by chemotherapy and manual vacuum extraction. Combined treatment modalities were used among 6.8 % of patients (Table 3).

**Table 3:** Presenting complaints and subsequent management of molar pregnancies at BPKIHS, Nepal

Symptoms and management		Number (*n* = 204)	Percentage (%)
Symptoms (multiple responses)	Abnormal uterine bleeding	176	86.3 %
	Lower Abdominal Pain	69	33.8 %
	Hyperemesis	54	26.5 %
	Passage of grape like cysts	24	11.8 %
	Hemoptysis	14	06.9 %
	Mass per abdomen	08	03.9 %
	Shortness of breath	06	02.9 %
	Fever	06	02.9 %
	Nausea and Vomiting	03	01.5 %
	Chest pain	02	01.0 %
	Abdominal distension	01	0.5 %
	Shock	01	0.5 %
	No symptoms	28	13.7 %
Management methods	Suction and Evacuation	183	89.8 %
	Suction and Evacuation + Chemotherapy	12	5.8 %
	Chemotherapy	5	2.4 %
	Spontaneous abortion	1	0.5 %
	Manual vacuum extraction	1	0.5 %
	Suction and Evacuation + Manual Vacuum Extraction	1	0.5 %
	Manual vacuum extraction + Chemotherapy	1	0.5 %

Table 4 shows that 56.4 % of patients suffered complications during management of cases. Blood transfusion was required among 45.6 % of patients, and anemia was seen among 40.2 % of the patients. There were 11.8 % were admitted in Intensive Care Unit (ICU) for post management care, fever was seen among 6.4 % of cases and Gestational Trophoblastic Neoplasia (GTN) was diagnosed in 5.9 % of cases.

**Table 4:** Complications (multiple responses) seen during and after management of molar pregnancies at BPKIHS, Nepal

Complications	Number	Percentage
Present	115	56.4 %
Absent	89	43.6 %
1. Blood transfusion	93	45.6 %
2. Anemia	82	40.2 %
3. Admission in ICU	24	11.8 %
4. Fever	13	6.4 %
5. Gestational Trophoblastic Neoplasia	12	5.9 %
6. Hemoptysis	10	4.9 %
7. Retain products of conception	06	2.9 %
8. Shortness of breath	05	2.5 %
9. Re-Manual Vaccum Aspiration	05	4.5 %
10. Edema	01	0.5 %
11. High blood pressure	01	0.5 %
12. Intravenous Iron therapy	01	0.5 %
13. Hyperthyroidism	01	0.5 %
14. Upper Respiratory tract Infection	01	0.5 %
15. Rise in Hcg	01	0.5 %
16. Septic abortion	01	0.5 %
17. Glossitis	01	0.5 %
18. Exploratory Laporotomy	01	0.5 %
19. Ventillation support	01	0.5 %
20. Invasive Mole	01	0.5 %

## Discussion

The 5- year prevalence of molar pregnancy at BPKIHS from 2008–2012 was 4.17 per 1000 live birth with range of annual prevalence of molar pregnancies during the study period between 3.85–4.50 per 1000 live births. A study reports a lower prevalence among Asian population of about 2.58 per 1000 live births [14]. As BPKIHS is the tertiary referral hospital for the whole of Eastern Nepal, the cases and the deliveries represent a large proportion of cases that show up at a hospital for treatment. However the prevalence may be different, as deliveries also happen at home or at birthing centers at governmental and private health institutions in eastern Nepal. Half of the delivery takes place at home in Nepal [15].

There are wide geographical variations in the incidence of gestational trophoblastic disease as a result of differences in methodology, classifications of mole, case detection [14]. Similarly, the epidemiology of trophoblastic disease in Nepal remains unknown as there are inconsistent findings from many centers. A study done from maternity hospital of Kathmandu reported annual incidence of 2.84 and 3.24 per 1000 live births while in another teaching hospital the incidence of trophoblastic disease ranged from 7.07 per 1000 pregnancies to 8.04 per 1000 deliveries [16]. A study done to compare GTD among Asian women of North England and North Wales showed that Asian women are at increased risk of having molar pregnancies compared to the western populations [14].

While in the current study, more than one third of the patients were in the age group of 20–35 years with a range of 16–51 and mean age of 23.7 years. Prevalence of molar pregnancy was found to be higher in women younger than 29 years (80 %) in another 8 year retrospective study done in Kathmanudu [9]. In other studies, it has been found that there is a relationship between risk of molar pregnancy and both upper and lower extremes of maternal age. Furthermore, the extent of risk is much greater with older rather than younger maternal ages, and it is only at the true extremes of maternal age (15 and 45 years) that the increase in risk sharply rises [17]. There is a need to look further about the association of age with molar pregnancies in future studies.

More than one third (41.7 %) of the patients were primigravida and about ten per cent gave a positive past history of molar pregnancy. In a descriptive case series from 2001 to 2007 in Bharatpur, Nepal showed that in 15.5 % of cases of molar pregnancy had occurred among primigravida and same proportion had positive past history of molar pregnancy [18] while large proportion (36.7 %) of primigravida suffered from molar pregnancy in another study [2]. However, studies have also shown that there is no real association of gravidity with molar pregnancy when corrected for age [19].

The period of gestation ranged from 8 to 34 weeks with a major proportion (66.4 %) having gestational age at time of evaluation more or equal to 13 weeks and the average uterine size at evaluation was 17.83 ± 6.54 weeks in the present study. A study from Sweden also reported the mean gestational age at the time of USG was 12.4 weeks similar to our study [20] while dissimilar findings was seen from the New England Trophoblastic Disease Centre [21]. According to their report, the mean estimated gestational age at evaluation was 11.8 weeks (range 6–22) and the mean uterine size at evaluation was 12.4 weeks (range 7–20 weeks) [21].

The mean level of pre-evacuation hCG of our patients was 18,644 IU/ml and after the management during the follow up after 1 week was 4156.84 IU/ml. This level was much lower compared to an Israeli study which reported The mean pre-evacuation β-hCG was 2,75,901 IU/l (range 2,011,000–9,19, 000 IU/l) [22]. The analysis of Hungarian patients in 2004 with uncomplicated hydatidiform mole, indicates that once undetectable serum hCG levels are attained relapse is unlikely. The follow up of uncomplicated partial moles and complete moles with weekly serum hCG levels until negative titers seems to be safe [23].

The USG findings among patients attending BPKIHS suggests that more than one fifth (20.6 %) of pregnant women had theca lutein cyst. USG service is available in some institutions of eastern Nepal. However, there are limited places in referral area with reliable recording and reporting done by ultrasonologists. Similar proportion (24.5 %) of theca lutein was reported by a study in another region of Nepal [18].

The patients presented to our center with abnormal uterine bleeding as the most frequent (86.3 %) complaint. The other presenting symptoms were pain (33.8 %), hyper emesis (26.5 %) and passage of grape like cysts (11.8 %) while 13.7 % did not report any symptoms and were diagnosed during routine examination. More than one third (38.7 %) of patients had pallor on examination.

Similar complaints were reported by patients with molar pregnancies in various studies. Vaginal bleeding was the most common presenting symptom while presence of excessive uterine size, anaemia, pre-eclampsia, hyperemesis and hyperthyroidism was significantly less common among current patients than in past cases at a centre [21].

A study from Israel showed that although vaginal bleeding was the most common presenting symptom while 41 % of their patients were asymptomatic. Furthermore, systemic manifestations such as hyperemesis, pre-eclampsia, clinical thyrotoxicosis and respiratory distress were exceedingly rare in this study [22]. It was seen from reports of a study done in Sweden that the current clinical presentation of complete mole has clearly changed compared to that of the classic type of mole with vaginal bleeding (77 %), abdominal pain (23 %) and hyperemesis (19 %) being the most commonly occurring symptoms. The clinical presentation of partial moles usually includes no typical symptoms. Rather, the signs and symptoms are those of incomplete abortion or missed abortion [23].

In the present study, the various management methods were suction evacuation, chemotherapy and manual vacuum extraction. Combined treatment modalities were used among 6.8 % of patients. At another center in Nepal, 13.3 % of patients were treated with suction evacuation, 62.2 % of patients underwent adjuvant chemotherapy among which 26.6 % received single agent chemotherapy and received EMA-CO regimen [18].

Gestational Trophoblastic Neoplasia (GTN) was diagnosed in 5.9 % of cases the cases could suggest that there may be over diagnosis of molar pregnancy in this center. This may need further investigation in the future.

A large proportion (56.4 %) of the patients in this study required extra attention and suffered some form (major or minor) complications during management of cases. This can be due to patients coming with more severe conditions and complaints in which management is aggressive and require long term attention and care. With anemia seen among 40.2 % of the patients, transfusion was done in 45.6 % of the patients. A high level of is reported by another study [21]. ICU admission and respiratory distress have been reported in other studies [24–26]. During the management of molar pregnancy, patients are required to pre arrange blood for transfusion. While those with anticipated hemorrhage are transfused, many don’t require transfusion. Admissions in ICU included those patients with shortness of breath, septic abortion, exploratory laporotomy, patient requiring ventilation support, and those that are anticipated to required were kept in Intensive unit for observation for 24 to 48 h. However, since these are based on the medical records the practices could not be further explored in detail. There seems to be a need for a standard protocol for management of molar pregnancy in our hospital.

This study used a secondary data source and thus, validity and reliability of the data is of concern. Patients of molar pregnancy need follow up for at least 9 months, there was no data on follow up of patients which also an important issue. The study has limitations of not having the descriptions of the type of molar pregnancy. The medical records could not provide the findings dings on the histopathologic examinations (HPE) which could have given a more clearepicture molar pregnancy. There is a need to strengthen the recording and reporting system to include the comprehensive records in our hospital.

## Conclusion

The 5-year prevalence of molar pregnancy at B.P. Koirala Insitute of Health Sciences—the only tertiary care center of eastern Nepal during the study period of 2008–2012 is 4.17 per 1000 live births. Similar studies in the future at a national level will help us to reach a national figure regarding prevalence and incidence of molar pregnancies in Nepal.
